# Catecholamine-Induced Secondary Takotsubo Syndrome in Children With Severe Enterovirus 71 Infection and Acute Heart Failure: A 20-year Experience of a Single Institute

**DOI:** 10.3389/fcvm.2021.752232

**Published:** 2021-09-23

**Authors:** Sheng-Ling Jan, Yun-Ching Fu, Ching-Shiang Chi, Hsiu-Fen Lee, Fang-Liang Huang, Chung-Chi Wang, Hao-Ji Wei, Ming-Chih Lin, Po-Yen Chen, Betau Hwang

**Affiliations:** ^1^Department of Pediatrics, Children's Medical Center, Taichung Veterans General Hospital, Taichung, Taiwan; ^2^Department of Pediatrics, School of Medicine, National Yang-Ming University, Taipei, Taiwan; ^3^Department of Pediatrics, School of Medicine, Kaohsiung Medical University, Kaohsiung, Taiwan; ^4^Department of Pediatrics, Tungs' Taichung Metroharbor Hospital, Taichung, Taiwan; ^5^Department of Cardiovascular Surgery, Cardiovascular Medical Center, Taichung Veterans General Hospital, Taichung, Taiwan

**Keywords:** catecholamine, enterovirus 71, heart failure, dilated cardiomyopathy, Takotsubo syndrome

## Abstract

**Background:** Acute heart failure (AHF) is the major cause of death in children with severe enterovirus 71 (EV71) infection. This study aimed to report our clinical experience with EV71-related AHF, as well as to discuss its pathogenesis and relationship to Takotsubo syndrome (TTS).

**Methods:** A total 27 children with EV71-related AHF between 1998 and 2018 were studied. The TTS diagnosis was based on the International Takotsubo Diagnostic Criteria.

**Results:** Acute heart failure-related early death occurred in 10 (37%) of the patients. Sinus tachycardia, systemic hypertension, and pulmonary edema in 100, 85, and 81% of the patients, respectively, preceded AHF. Cardiac biomarkers were significantly increased in most patients. The main echocardiographic findings included transient and reversible left ventricular (LV) regional wall motion abnormality (RWMA) with apical ballooning. High concentrations of catecholamines either preceded or coexisted with AHF. Myocardial pathology revealed no evidence of myocarditis, which was consistent with catecholamine-induced cardiotoxic damage. Patients with EV71-related AHF who had received close monitoring of their cardiac function, along with early intervention involving extracorporeal life support (ECLS), had a higher survival rate (82 vs. 30%, *p* = 0.013) and better neurological outcomes (59 vs. 0%, *p* = 0.003).

**Conclusion:** EV 71-related AHF was preceded by brain stem encephalitis-related hypercatecholaminemia, which resulted in a high mortality rate. Careful monitoring is merited so that any life-threatening cardiogenic shock may be appropriately treated. In view of the similarities in their clinical manifestations, natural course direction, pathological findings, and possible mechanisms, TTS and EV71-related AHF may represent the same syndrome. Therefore, we suggest that EV71-related AHF could constitute a direct causal link to catecholamine-induced secondary TTS.

## Introduction

Most children infected by enterovirus 71 (EV71) develop either hand-foot-mouth disease (HFMD) or herpetic angina. Less than 0.01% of the patients are complicated by central nervous system (CNS) involvement, pulmonary edema, acute heart failure (AHF), shock, or early death (1–5). Our previous study found that EV71-related early deaths are usually accompanied by acute left ventricular (LV) dysfunction and LV regional wall motion abnormality (RWMA) (4–11). Therefore, AHF has been proposed as the major cause of early death in these patients (4–12), with EV-71 brainstem encephalitis-related hypercatecholaminemia believed as having a direct impact on the cardiotoxicity leading to AHF (7, 11, 13). Jan and Fu et al. found that patients with severe EV71 infection and AHF have clinical manifestations of sympathetic hyperactivity and significantly increased catecholamine concentrations. This result supports the hypothesis that hypercatecholaminemia is the cause of EV-71 related AHF (7, 11).

Takotsubo syndrome (TTS) was first reported in Japan in 1990 (14). The condition is characterized by reversible LV systolic dysfunction, which has a unique pattern similar to RWMA occurring in acute coronary syndrome (ACS), but without occluded coronary arteries, thus explaining the pattern of transient LV dysfunction. The syndrome is usually preceded by an emotional or a physical stress factor (15, 16). Although TTS is generally presented as a benign disease, serious complications, including AHF and cardiogenic shock, can lead to increased mortality. The reported incidence of cardiogenic shock in TTS patients ranges from 6 to 20% (15). Takotsubo syndrome most commonly occurs in postmenopausal women (15, 16), but is an uncommon condition in children (17, 18). The pathogenesis of TTS remains largely uncertain. Catecholamine-mediated cardiac stunning may be the main cause (15, 16, 19–21). The pathophysiology of TTS appears to be similar to the hypothesis of hypercatecholaminemia-related AHF in severe EV71 infection. In the present study, we describe the first study performed on children with severe EV71 infection and AHF, while presenting a clinical picture resembling TTS. A review of our previous studies is presented, with those findings shedding light on the pathophysiological concepts involved in this novel cardiac syndrome affecting children with severe EV71 infection.

## Methods

### Study Population

Data from a total of 27 children, each with a laboratory-confirmed clinical diagnosis of severe EV71 infection and AHF from the years 1998 to 2018, were collected. Viral identification was also confirmed by the Taiwan Centers for Disease Control. The clinical features of EV71 infection have been divided into four stages and modified from the previous study (22). Stage 1, HFMD or herpangina without complication; Stage 2, CNS involvement with encephalomyelitis; Stage 3, Autonomic dysregulation; and Stage 4, Acute heart failure, including acute LV systolic dysfunction and shock. Severe EV71 infection was defined as EV71 infection beyond Stage 2. Patients suffering from neuromuscular diseases, craniofacial anomalies, genetic diseases, or previous cardiovascular diseases, as well as those who could not participate due to other reasons were excluded. Amongst the 27 children with severe EV71 infection and AHF (Stage 4), 10 received only conventional medical treatment between 1998 and 2000, and were defined as the pre-ECLS era cohort. Seventeen of the 27 patients experienced a poor response to conventional medical treatments and were rescued through extracorporeal life support (ECLS) between 2000 and 2018. These 17 were defined as the post-ECLS era cohort.

### Diagnostic Imaging Study

Chest X-ray and a complete transthoracic echocardiography, using the Philips Sonos 5500, 7500 or iE33 ultrasound system (Philips, Andover, Massachusetts, USA), were performed on all patients within half an hour of entering the pediatric intensive care unit (PICU), and repeated as needed. Key echocardiographic features in this study consisted of LV systolic dysfunction and circumferential RWMA involving the mid-ventricular segments. If RWMA existed, the ejection fraction (EF) was measured using the biplane Simpson's rule. Possible causes of ventricular dysfunction, such as congenital coronary artery anomalies and severe ventricular outflow obstruction, were excluded. All echocardiographic studies were performed by experienced pediatric cardiologists (S-LJ, Y-CF, M-CL) (4–11).

### Myocardial Histology Study

Myocardial histology using the elastic tissue-Masson technique, hematoxylin and eosin stain, and *in situ* terminal deoxyribonucleotidyl transferase-mediated dUTP nick end labeling assay were all studied. All myocardial specimens were sent for an enterovirus culture and analyzed by real-time polymerase chain reaction (7).

### Cardiac Biomarkers Study

Laboratory data including complete blood count, blood glucose, biochemical tests, C-reactive protein, and cardiac biomarkers such as creatinine kinase (CK), muscle-brain fraction of creatine kinase (CK-MB), cardiac troponin I, B-type natriuretic peptide (BNP), or N-terminal prohormone of BNP (NT-proBNP) were studied (4–11). Hyperglycemia was defined as being a blood glucose concentration over 150 mg/dL on admission to the PICU. The upper limits of normal were 150 units/L for CPK, 5% for CK-MB, 1 ng/ml for cardiac troponin I, 100 pg/ml for BNP, and 125 pg/ml for NT-proBNP.

### Catecholamine Study

Blood samples were collected upon admission to the PICU, and with the plasma stored at −70°C until analysis. After collection, urine samples were protected from light and stored at 4°C. They were then acidified with concentrated hydrochloric acid and stored at −20°C until analysis. Measured epinephrine (Epi), norepinephrine (NE), and vanillylmandelic acid (VMA) samples were taken using commercially available high-performance liquid chromatography, combined with the electrochemical detection method (7, 11). We corrected the relative concentration of urine catecholamine and VMA based on urine creatinine concentration and then expressed it as a ratio to the creatinine concentration (7, 11).

### Diagnosis of TTS

The TTS diagnosis in this study was mainly based on the international Takotsubo diagnostic criteria (19), including (1) acute onset of symptoms; (2) transient RWMA with acute LV dysfunction by transthoracic echocardiography; (3) elevation in levels of cardiac biomarkers, CK-MB, cardiac troponin I, BNP, or NT-proBNP; (4) no evidence of infectious myocarditis; and (5) complete normalization of wall motion abnormality and LVEF, except in patients who died prior to evidence of recovery being captured.

### Statistical Analysis

The data was displayed as the numbers of the case, percentage, median, or mean ± standard deviation. *SPSS, Version 22.0 for Windows* (SPSS, Chicago, IL, USA) was used for statistical analysis. The Mann-Whitney *U*-test, Pearson's Chi-square test, or Fisher's exact test were used to compare clinical manifestations, laboratory features, and outcomes between the pre-ECLS era and post-ECLS era cohorts. The Kaplan-Meier method was used to analyze the survival rate of EV71-related AHF patients. A two-tailed test result of *P* < 0.05 was considered statistically significant.

## Results

### Patient Profiles

**Figure 1:**
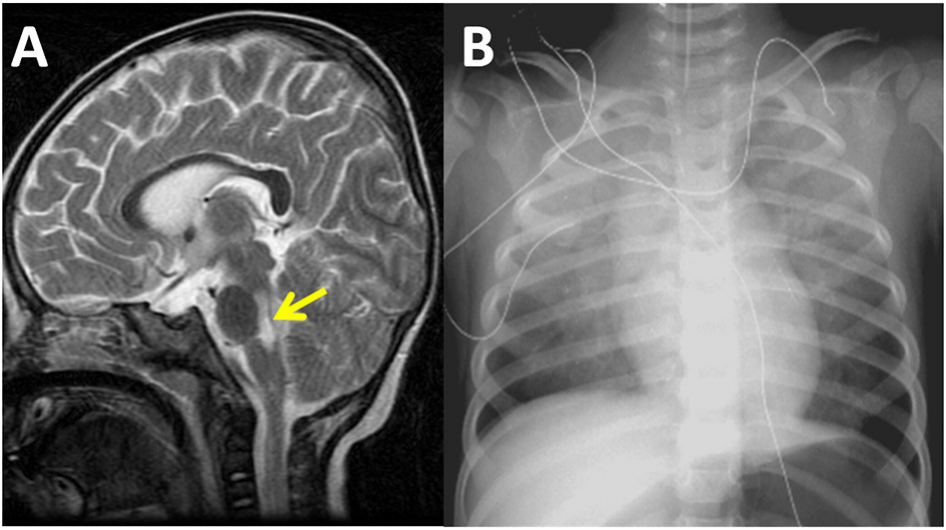
Sagittal magnetic resonance imaging of the brain reveals high signal intensity over the dorsal regions of the brain stem and cervical spinal cord in a 16-month-old patient with severe enterovirus 71 infection and acute heart failure in the year 2001 **(A)**. Chest radiography demonstrates bilateral pulmonary edema and a normal heart size. The ejection fraction of the left ventricle was 27% as measured by echocardiography at this time **(B)**.

The demographic data, clinical manifestations, and outcomes of the patients are shown in Table 1. The median age of the patients was 17 months (4–69 months), with the male/female ratio being 1.7. Enterovirus 71-related AHF occurred in patients 2–4 days (median of 3 days) after EV71 infection symptoms were noted. All 27 patients experienced brainstem encephalitis (Figure 1A) and sinus tachycardia, with their maximum heart rate ranging from 185 to 260 bpm. Twenty-three (85%) patients on arrival to the PICU had preexisting systemic hypertension, while hypotension was diagnosed in 4 (15%), if cardiogenic shock occurred. Comparing the cohorts between the pre-ECLS era and post-ECLS era, there were 7 (70%) and 3 (18%) patients who experienced early death due to AHF, while three (30%) and 1 (6%) experienced late death due to encephalopathy, respectively. Compared with the pre-ECLS era cohort, patients in the post-ECLS cohort had better neurological outcomes (good neurological outcomes of 59 vs. 0%, *p* = 0.003), as well as higher survival rates (82 vs. 30%, *p* = 0.013) (5, 7, 8).

**Table 1:** Comparison of cohort data from before and after the era of using ECLS to rescue severe EV71-related acute heart failure.

	**Total**	**Pre-ECLS era**	**Post-ECLS era**	***P* value**
	**years (1998–2018)**	**(1998-2000)**	**(2000-2018)**	
**Variables**	**(*n* = 27)**	**(*n* = 10)**	**(*n* = 17)**	
**Clinical Data**				
Age (months)	21 ± 16 (17)	18 ± 14 (13)	23 ± 17 (20)	0.505
Gender	17 M/10 F	5 M/5 F	12 M/5 F	0.415
BW (kg)	11 ± 4 (11)	10 ± 3 (10)	12 ± 5 (11)	0.334
Onset to admission (days)	4 ± 1 (3)	4 ± 1 (4)	3 ± 1 (3)	0.243
MaxHR (bpm)	206 ± 28 (205)	204 ± 37 (209)	207 ± 22 (200)	0.980
MaxSBP (mmHg)	119 ± 27 (123)	117 ± 15 (118)	120 ± 32 (131)	0.141
**Diagnostic Imaging**				
CTR in CxR	0.50 ± 0.04 (0.52)	0.50 ± 0.06 (0.53)	0.50 ± 0.04 (0.52)	0.824
Pulmonary edema	22 (81%)	9 (90%)	13 (76%)	0.621
LVEDD, Z-score	1.2 ± 2.1 (0.8)	0.9 ± 1.9 (1.0)	1.3 ± 2.2 (0.8)	0.639
Initial EF%	36 ± 11 (36)	38 ± 14 (36)	35 ± 9 (36)	0.570
RWMA	23 (85%)	8 (80%)	15 (88%)	0.613
**Laboratory Data**				
CK (IU/L)	330 ± 297 (344)	344 ± 445 (183)	323 ± 168 (369)	0.201
CK-MB (IU/L)	65 ± 237 (20)	135 ± 383 (11)	23 ± 10 (22)	0.182
Glucose (mg/dl)	268 ± 228 (199)	251 ± 137 (232)	276 ± 267 (140)	0.525
**Intervention and Outcomes**				
Rescued by ECLS	15 (56%)	0 (0%)	15 (88%)	0.000
Good neurological outcome	10 (37%)	0 (0%)	10 (59%)	0.003
Survival rate (>7 days)	17 (63%)	3 (30%)	14 (82%)	0.013
	10 early deaths	7 early deaths	3 early deaths	
	4 late deaths	3 late deaths	1 late death	

*Data are presented as mean ± standard deviation (median) or case numbers (%). BW, body weight; CK, creatine kinase; CK-MB, muscle-brain fraction of creatine kinase; CTR, cardiothoracic ratio; CXR, chest radiography; ECLS, extracorporeal life support; EF, ejection fraction of the left ventricle; EV71, enterovirus 71; MaxHR, maximum heart rate; LVEDD, left ventricular end-diastolic dimension; MaxSBP, maximum systolic blood pressure; RWMA, regional wall motion abnormalities. Good neurological outcome is defined as survival to discharge with a Pediatric Cerebral Performance Category Scale of 1, 2, or 3 upon hospital discharge. Comparison of data between pre-ECLS era and post-ECLS era cohorts were analyzed by the Mann-Whitney U-test, Pearson's Chi-square test, or Fisher's exact test*.

### Diagnostic Imaging Study

**Figure 2:**
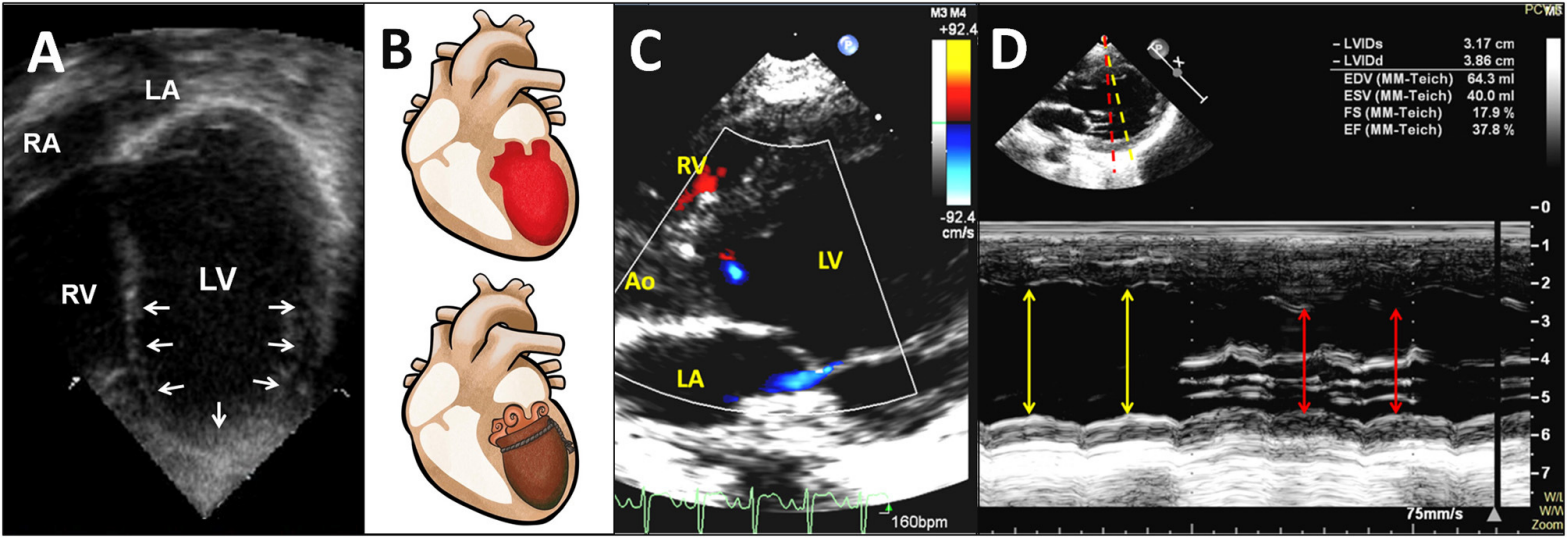
Transthoracic two-dimensional echocardiogram in the apical four-chamber view shows a mid-ventricular pattern of a circumferential regional wall motion abnormality with conspicuous apical ballooning of the LV in a 20-month-old patient in the year 2000 **(A)** (also see Supplementary Video 1). Diagram demonstrates the apical ballooning shape of the LV with an appearance resembling an octopus pot (called Tako-tsubo) **(B)**. Transthoracic color Doppler echocardiogram in the parasternal long-axis view shows LV dysfunction with a circumferential regional wall motion abnormality resulting in apical ballooning of the LV during systole, and mild mitral regurgitation in a 3-year-old patient in the year 2018 **(C)** (also see Supplementary Video 2). An M-mode echocardiographic recording from the level of the mitral valve chordae to the level of the mitral valve reveals a circumferential LV regional wall motion abnormality along yellow and red lines **(D)**. Ao, aorta; LA, left atrium; LV, left ventricle; RA, right atrium; RV, right ventricle.

The cardiothoracic ratios measured on chest radiographs ranged from 0.39 to 0.55 (median 0.52). In the chest X-rays of all patients, heart size was normal for age. Twenty-two (81%) patients developed pulmonary edema (Figure 1B). The M-mode z-score values of LV end-diastolic diameter ranged from −2.1 to 4.6 (median of 0.84), which was consistent with the normal heart sizes shown on chest radiography. Initial abnormal LVEF ranged from 17 to 46% (median of 36%) by echocardiography. There were 23 (85%) patients who had typical RWMA and 4 (15%) with late stage AHF who had diffuse LV akinesia (Figures 2A–D; Supplementary Videos 1, 2).

### Myocardial Histology Study

Histological examination of the myocardium of seven patients revealed that all myocardial specimens demonstrated obvious myofibrillar degeneration with coagulative myocytolysis, with most of the nuclei showing significant pyknosis and irregular shape. Myofibrillar waving, dissolution, and different degrees of cardiomyocyte apoptosis could be observed. The LV was more remarkably involved than the right ventricle. Through either viral culture, real-time polymerase chain reaction for virus detection or inflammatory cell infiltration, all heart specimens showed no evidence of viral myocarditis (7).

### Cardiac Biomarkers and Catecholamine Study

**Figure 3:**
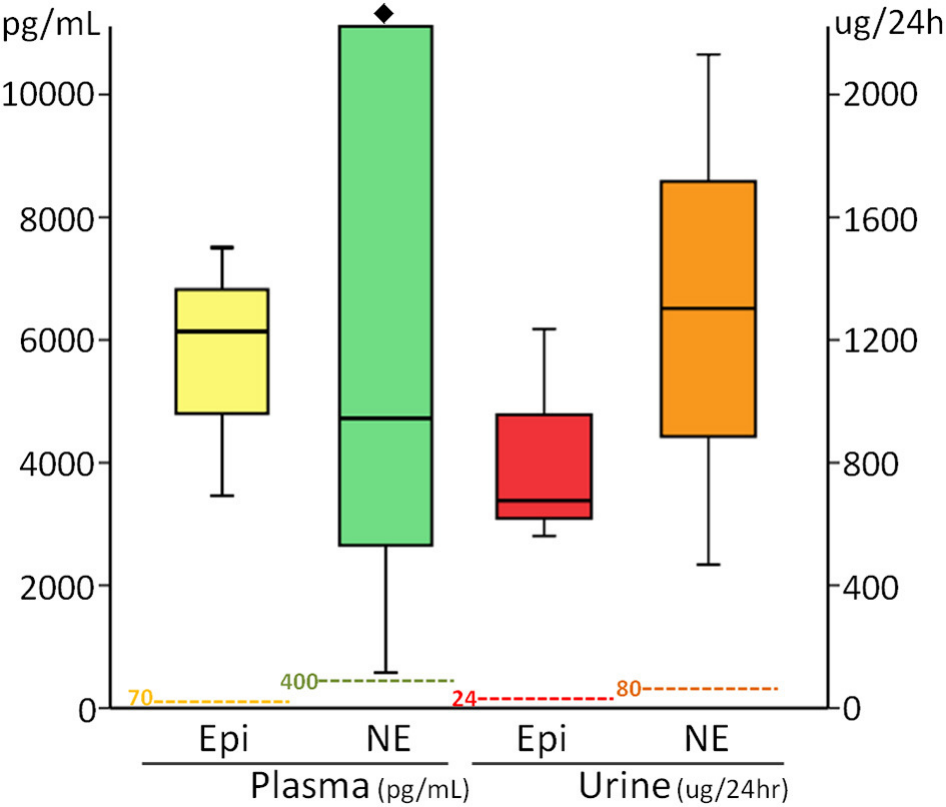
Box-plots show significant elevation of plasma and urine catecholamine concentrations in patients with enterovirus 71-related heart failure. Dash lines indicate individual upper limits of normal. ♦, Outliers: the patient's values that are between 1.5 and 3 box lengths from either end of the box. Epi, epinephrine; NE, norepinephrine.

Laboratory findings revealed hyperglycemia, elevated CK, CK-MB, cardiac troponin I, BNP, and NT-proBNP in 15 (56%), 16/26 (62%), 24/26 (92%), 7/8 (88%), 4/4 (100%), and 4/4 (100%) patients, respectively. The cardiac biomarkers CK-MB, cardiac troponin I, BNP, and NT-proBNP were 65 ± 237 IU/L (median of 20), 3.94 ± 2.77 ng/ml (median of 1.48), 296 ± 162 pg/ml (median of 238), and 3,956 ± 2,908 pg/ml (median of 3,095), respectively. Catecholamines were measured in SIX patients, including plasma catecholamines which were measured in three patients who showed high concentrations, with their Epi levels being 3,461, 7,505, and 6,137 pg/ml (normal range <70 pg/ml), and NE levels at 4,723, 54,418, and 579 pg/ml (normal range 100–400 pg/ml). Urine catecholamines were measured in three patients who displayed high Epi concentrations levels of 561, 677, and 1,236 μg/24 h (normal range 0–24 μg/24 h), NE levels of 1,303, 467, and 2,131 μg/24 h (normal range 10–80 μg/24 h), and VMA levels of 37.6, 14.2 and 28.4 mg/24 h (normal range 1–7 mg/24 h), respectively (Figure 3). Patients with EV71-related AHF had significantly higher troponin I, CK-MB, BNP, NT-proBNP, and catecholamine levels than those of patients without AHF. Using the cut-off values of BNP >100 pg/ml, urine Epi >134 μg/gCr, and urine NE >176 μg/gCr to identify EV71-infected patients with AHF, their sensitivity and specificity were both 100% for all when compared with those patients without AHF (7, 10, 11).

### Diagnosis of TTS

An acute onset of symptoms and acute LV dysfunction occurred in all patients. Echocardiographic findings with typical RWMA met the diagnosis criteria in 23 (85%) patients, whereas four patients with diffuse LV akinesia, possibly due to a late stage of AHF upon arrival, did not meet the diagnostic criteria. There was insufficient laboratory data seen in both cardiac biomarkers and histological examination of the myocardial to allow for a full analysis, but the available parameters were elevated in most of the patients. There was no evidence of infectious myocarditis in 7 of 7 (100%) patients, while BNP and NT-proBNP both significantly increased in 4 of 4 (100%) patients. In 17 of the 27 patients, there was complete normalization of wall motion abnormality and LVEF, although early death occurred in the other 10 patients prior to complete normalization of LVEF. Most of the recovery time of LV systolic function was about 3–5 days.

## Discussion

Children with enterovirus infection are more likely to experience no complications, except when the pathogen is EV71. Enterovirus 71 was first discovered in California patients in 1974, where they were presented with serious neurologic complications. The virus is related to sporadic cases and outbreaks, and is distributed globally. Countries including Bulgaria in 1975 (44 deaths), Hungary in 1978 (47 deaths), Malaysia in 1997 (more than 31 deaths), and Taiwan in 1998 (78 deaths) have all been noted (1–3, 7). Brain stem encephalitis was the main neurological complication and was present in all of the fatal EV71 infection cases (1–3). However, AHF was the main cause of early mortality (4–9). All patients ran a similar fulminant course resulting in death. After experiencing several days of HFMD symptoms, herpangina, or febrile illness (Stage 1), patients developed brain stem encephalitis (Stage 2), with some developing sympathetic hyperactivity (Stage 3), and a few progressing to AHF with or without pulmonary edema (Stage 4), shock, and even rapid death, despite intensive management attempts (1–5).

### EV71 Encephalitis, Hypersympathetic Activity, and Hypercatecholaminemia

Although hypersympathetic activity is preceded by CNS involvement, its pathophysiology remains unclear. The main pathological finding in EV71 encephalomyelitis is CNS inflammation, predominantly involving the whole brain stem and gray matter of the spinal cord. The lesions in these regions can increase hypersympathetic activity, and may result in peripheral vascular constriction, diaphoresis, tachycardia, and systemic hypertension, with surges of hypercatecholaminemia (13). In previous studies, we proved that there were significantly high concentrations of catecholamines in patients with EV71-related AHF which was preceded by hypersympathetic symptoms (7, 10, 11).

### EV71-Related AHF

Based upon our prior knowledge and observations of the clinical manifestations, the characteristic presentations of patients with EV71-related AHF seem to include several issues. First, typical echocardiographic findings involved a mid-ventricular pattern of circumferential LV RWMA with apical ballooning, known as “panic or shivering heart”, as well as acute LV dysfunction. Second, pulmonary edema and a normal heart size were noted in most patients experiencing heart failure, which has been termed, “heart function-chest radiograph dissociation.” If only chest radiographs are used for assessment, this phenomenon may lead to unrecognized cardiac dysfunction in these patients. Third, there was no evidence of infectious myocarditis through patients' histology. Fourth, sympathetic hyperactivity and high concentrations of catecholamines were either followed by or coexisted with LV dysfunction. Fifth, most patients had a significant increase in cardiac biomarkers, indicating a certain degree of cardiac damage. Sixth, myocardial pathology results possibly indicated catecholamine-induced cardiac damage. Seventh, LV dysfunction was both acute and transient, but also usually fatal. Eighth, shock patients with hypotension more often experienced rapid clinical deterioration and adverse neurological outcomes. Ninth, short-term ECLS support treatment for transient cardiac dysfunction caused by catecholamine storm had a lower mortality rate and fewer neurological sequelae.

### EV71-Related AHF and TTS

Catecholamines seem to play an important role in the pathogenesis of TTS, reflecting the comprehensive response of the cardiovascular system to either a sudden increase in the concentration of endogenous catecholamines, which is usually related to acute severe stress, or exogenous catecholamines (15, 16, 19–21). Cases can be classified as primary or secondary TTS (23). Biopsy samples obtained from patients in the acute phase of TTS are similar to those resulting from the direct effects observed after catecholamine-induced cardiotoxicity (16). Evidence from clinical studies supports the hypothesis that excess catecholamine serves as triggers for TTS (15, 16, 19–21). Paur et al. (24) reported an animal study concerning TTS that revealed a high epinephrine concentration can induce LV apical ballooning by injecting an exogenously high-dose of epinephrine. Fu et al. (25) developed a feline model of NE cardiotoxicity and compared it to children with EV71-related AHF. His study concluded that AHF in patients with EV71 encephalitis was similar to that in cats experiencing NE cardiotoxicity. In view of the similarities in clinical manifestations, natural course direction, pathological findings and possible pathogenesis, TTS and EV71-related AHF may represent the same syndrome. Therefore, we suggest that there is a direct causal link between EV71-related AHF and catecholamine-induced secondary TTS.

### Pathogenesis of EV71-Related TTS

**Figure 4:**
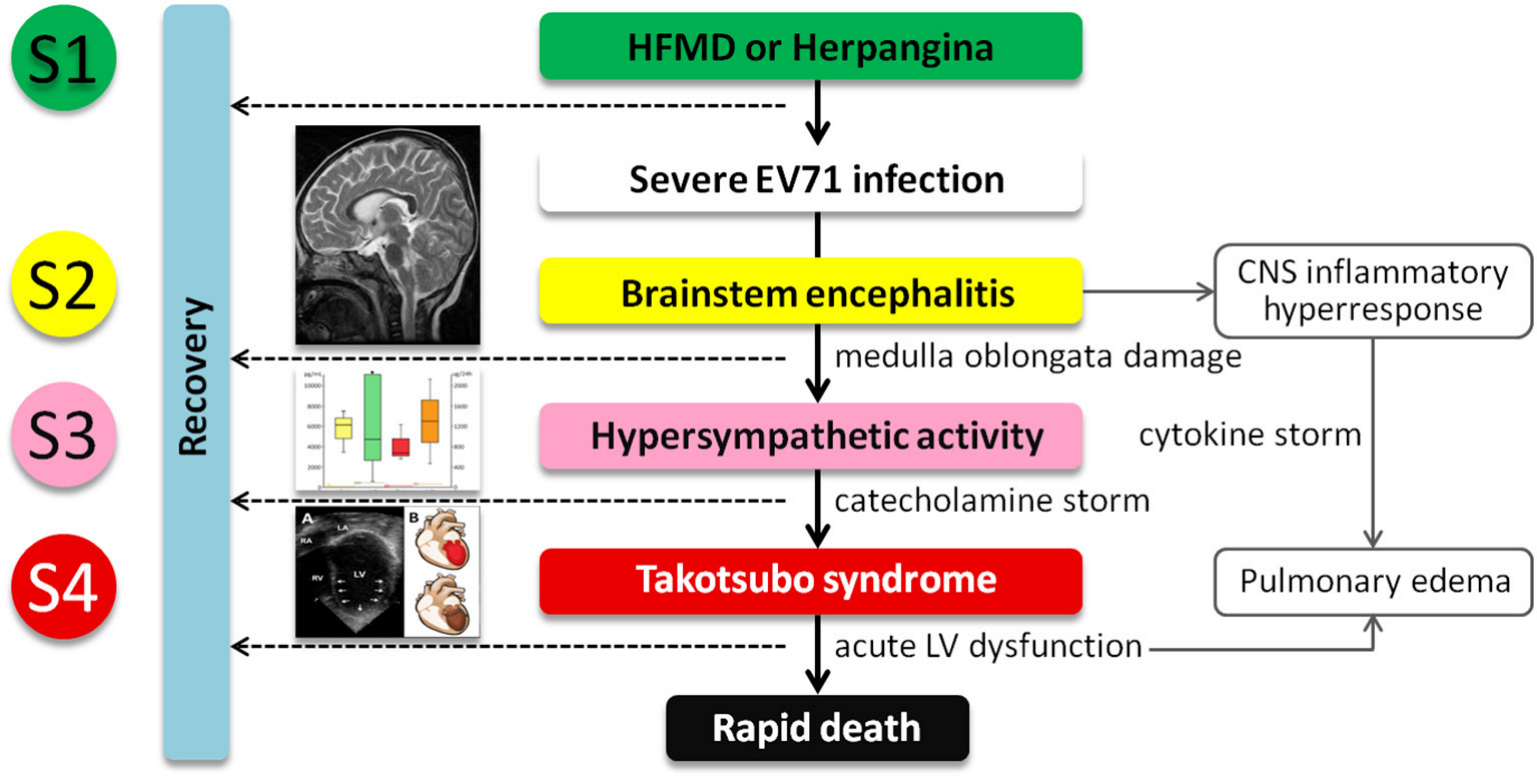
The postulated pathogenesis of severe EV71 infection associated with catecholamine-induced secondary Takotsubo syndrome (TTS). Central nervous system indicates central nervous system; EV71, enterovirus 71; HFMD, hand-foot-and-mouth disease; LV, left ventricular; and S1–4, Stage 1–4.

Our postulated pathogenesis of EV71-related TTS is presented in Figure 4. The pathophysiology of severe EV71 infection-related AHF and TTS can be generally divided into two phases. The first starts with an increased release of catecholamines, such as Epi and NE, initiated by EV71-related encephalitis, thus causing damage to the brain stem. The serum catecholamine concentration of severe EV71 patients is significantly higher at the time of AHF when compared with those in their non-AHF counterparts. These differences indicate that susceptible individuals may release excessive catecholamines. However, the thresholds for EV71-related encephalitis and excessive catecholamine release are incompletely understood. In recent years, several published reports have revealed the role of this brain-heart axis in the pathogenesis of TTS. Templin et al. (26) reported less functional connectivity in the limbic system of patients with TTS compared with healthy individuals. That region is important in emotional management and autonomic system regulation. Based on this reasoning, we consider that children with severe EV71 infection who develop TTS may have different autonomic regulation and the limbic system functions. The second phase is the cardiovascular reactions to the surge in circulating catecholamines. At this stage, catecholamine-induced TTS can be characterized by peripheral arterial vasoconstriction leading to an increase in afterload and transient high LV end-systolic pressure, acute coronary artery vasospasm resulting in both myocardial ischemia and a subsequent reduction in cardiac output with systemic hypotension, and finally catecholamine-mediated myocardial stunning which occurs directly at the apex where the β-adrenergic receptor gradient is highest. This area is highly sensitive to circulating catecholamines (16, 23). Despite the presence of hypercatecholaminemia, the development of hypotension may be the late stage of cardiogenic shock, or an aberrant response to catecholamine secretion, with the development of biased beta-2-mediated post-receptor signaling, and thus excessive release of the vasodilator nitric oxide (NO). Patients with TTS are hyper-responsive to NO, and in combination with the release (via catecholamines) of superoxide anion, generate peroxynitrite, a major inflammatory mediator. This “nitrosative stress” is responsible for damage to the glycocalyx lining the vasculature (for example, via plasma concentrations of the glycocalyx component SD-1), facilitating increases in vascular permeability. Thus, despite the high catecholamine levels, patients with TTS develop both hypotension (partially via NO effect, partially via volume depletion) and peripheral edema.

## Limitations

Due to the rarity of the condition, studies of children with severe EV71-related AHF are mostly limited to either case reports or series (3, 12). Although we have 20-years of experience treating children with EV71-related AHF at our institute, the case numbers in this study are limited. However, although similar clinical presentations have been found (3, 12), to the best of our knowledge this study is the largest case series in this field. Second, although there is currently a lack of non-invasive tools for a quick and reliable diagnosis of TTS, coronary angiography and left ventriculography are considered to be the gold standard diagnostic tools for confirming TTS. We lacked any evidence of absence of coronary artery disease (CAD) in this study, and also could not fully exclude any other conditions of secondary TTS, such as acute subarachnoid hemorrhage and pheochromocytoma. However, those conditions are uncommon in young children, and most published reports on TTS do not comply with these exclusion rules (19, 27). We cannot completely rule out the possibility that ACS-induced TTS may be the main event in our cohort, which in turn caused EV71 infection resulting in encephalitis and AHF. Nevertheless, the clinical manifestations (no history of heart disease predisposed to ACS prior to the event, and no myocarditis at autopsy) are more consistent with TTS being the initial precipitating event. Moreover, the prevalence of concomitant CAD in adult patients ranges from 10 to 29% (19). Therefore, the existence of CAD should not be regarded as an exclusion criterion, as acknowledged by International Takotsubo Diagnostic Criteria (19). Third, although cardiac magnetic resonance (CMR) with new parametric techniques has become an important tool for the non-invasive assessment of the TTS at the time of acute presentation and can help distinguish between TTS and other important differential diagnoses, such as myocarditis and myocardial infarction (28), we did not perform CMR for patients in this study due to all of them were hemodynamically unstable at the time of acute presentation. Fourth, we did not check cytokine profiles in this study. Cytokine storm can lead to AHF by IL-6-induced diastolic dysfunction and increased cardiomyocyte stiffness. Moreover, IL-1β and TNF-α produce negative inotropic effects and may induce cardiomyocyte pyroptosis and apoptosis, respectively (29, 30). However, there are limited data on cytokine storm-induced AHF (31, 32), and its clinical presentations and echocardiographic findings are not consistent with EV71-related AHF and TTS. Fifth, we did not perform specific immunohistological staining in this study. Immunohistological studies in patients with TTS and in rat models of TTS show that there is considerable inflammation, both cellular (leukocyte and macrophage infiltration) and humoral (thioredoxin-interacting protein), together with increased tissue 3-nitrotyrosine and poly(ADP-ribose) contents (33). Finally, the recognition of RWMA is subjective and involves substantial interoperative variability, despite all echocardiographic examinations being performed by experienced pediatric cardiologists in this study. Huang et al. (34) reported that automated interpretation of echocardiography by deep neural networks could be used to assist in the recognition of RWMA. Therefore, we consider that using these deep neural networks to automate the recognition of RWMA would be valuable in both supporting clinical reporting and improving efficiency in the future treatment of children with severe EV71 infection and AHF.

## Conclusion

Severe EV71 infection with AHF preceded by brain stem encephalitis-related hypercatecholaminemia has a high mortality rate. Enterovirus 71-related hypercatecholaminemia may serve as a trigger factor for TTS in the same manner as severe physical stress factors. Additionally, patients with EV71-related AHF can be considered to have secondary TTS, which manifests itself as a form of chemical and toxic cardiomyopathy. Obtaining a detailed medical history, closely monitoring vital signs and clinical symptoms, routine measurement of NT-proBNP and troponin concentrations, follow-up echocardiography, as well as follow-up CMR may all aid in the diagnosis of TTS in EV71 patients with AHF. Speckle tracking echocardiography should be used to monitor cardiac function, not just ejection fraction. They also need to be carefully monitored in order to treat any life-threatening cardiogenic shock which may occur at the appropriate time. Further research is still required in order to help clarify the hypotheses discussed herein and increase our understanding of the thresholds for excess catecholamine release and the cardiovascular responses to EV71-related encephalitis. These additional studies will also help the medical community better understand the pathophysiology underpinning severe EV71 infection-induced secondary TTS in young children.

## Data Availability Statement

The raw data supporting the conclusions of this article will be made available by the authors, without undue reservation.

## Ethics Statement

The studies involving human participants were reviewed and approved by The study complied with the Declaration of Helsinki, and all published studies were approved by the Institutional Ethics Committee of Taichung Veterans General Hospital. Written informed consent to participate in this study was provided by the participants' legal guardian/next of kin.

## Author Contributions

S-LJ and Y-CF contributed to the concept and design of this study, drafting of the manuscript, and statistical analysis. S-LJ, H-FL, F-LH, C-CW, and H-JW contributed to acquisition, analysis, and interpretation of the data. C-SC, M-CL, P-YC, and BH contributed to revision and finalize the manuscript. All authors contributed to the article and approved the submitted version.

## Conflict of Interest

The authors declare that the research was conducted in the absence of any commercial or financial relationships that could be construed as a potential conflict of interest.

## Publisher's Note

All claims expressed in this article are solely those of the authors and do not necessarily represent those of their affiliated organizations, or those of the publisher, the editors and the reviewers. Any product that may be evaluated in this article, or claim that may be made by its manufacturer, is not guaranteed or endorsed by the publisher.

## Supplementary Material

Supplementary Video 1 RWMA with apical ballooning of the LV in the four-chamber view.

Supplementary Video 2 RWMA with apical ballooning of the LV in the long-axis view.
